# Health anxiety and collective sense-making in the Chinese social media sphere: a topic modeling assessment of Weibo chatters

**DOI:** 10.1186/s12889-026-28505-8

**Published:** 2026-07-11

**Authors:** Lixia Peng, Zixue Tai, Xiaolong Liu

**Affiliations:** 1 School of Journalism and Communication, Minjiang University, 200, Xiyuangong Road, Fuzhou, 350108 Fujian Province China; 2 School of Journalism and Media, University of Kentucky, 507 Library Drive, Lexington, KY 40506 USA; 3 School of Marxism, Guangdong Pharmaceutical University, 280 Waihuan Road East, Guangzhou, 510006 Guangdong Province China

**Keywords:** Health anxiety, Extended Parallel Process Model, EPPM, Latent Dirichlet Allocation, LDA topic model, Content analysis, Weibo

## Abstract

**Background:**

The pervasive penetration of social media is reshaping the generative logic and communication pathways of health anxiety, which is significantly impacting public mental health and individual behavior. This study aimed to examine the topic threads of health anxiety discussions on Sina Weibo as well as the threat and efficacy elements contained in the health-related messages through the Extended Parallel Process Model (EPPM) framework.

**Methods:**

A total of 138,587 social media posts related to health anxiety were collected using keyword combination of “health” AND (“worry” OR “concern” OR “anxiety”) on the Weibo open API. Latent Dirichlet Allocation (LDA) model procedures were applied to identify the main topics in health anxiety discussions. Moreover, 1,480 most popular posts ranked by “Like” clicks were selected for manual textual analysis. Based on the Extended Parallel Process Model (EPPM), four dimensions—severity, susceptibility, self-efficacy, and response efficacy—were used as the coding framework for content analysis to systematically ascertain the topic structure, information characteristics and information engagement with health anxiety Weibo discussions.

**Results:**

Discussions of health anxiety cover a wide range of topics. The most prominent topic is positive living and health ideals (intensity = 0.4456), and other major topics include sleep disorders and mental health, adolescent mental health education and social adaptation, healthy lifestyle and self-management. There is a noticeable imbalance between elements of threat and efficacy in health anxiety discussions. Threat information dominates health anxiety discussions, with severity being the most prevalent component (73.24%). Conversely, efficacy information is much less featured, particularly regarding response efficacy (16.15%). Higher perceived threat levels in health anxiety discussions correlate with reduced engagement (fewer likes), whereas greater efficacy information levels are associated with increased engagement (more likes).

**Conclusions:**

Health anxiety discussions on social media exhibit a generative logic characterized by thematic threads emphasizing positive confidence-building, proactive measures, ailment-specific guidance, and subgroup-focused solution tips. Users’ conception of health anxiety reflects a contradictory pattern of “high threat-low efficacy”, wherein information about health threats is far more prevalent than information about self-efficacy. These findings show that EPPM-related threat and efficacy elements are distributed in user-generated Weibo discussions.

## Introduction

With steady improvement of living standards and rapid advancement of healthcare technology, we are witnessing a drastic shift in the medical paradigm from merely curing diseases to actively preventing them [1]. This new paradigm places great emphasis on a patient-centered approach in which patient engagement through a continuous care model can lead to sustained positive outcomes in patients’ physical and mental wellbeing [2]. This model of self-care has been greatly facilitated, and in many cases made possible, by the expansion and public use of a variety of digital health and information technologies. The advent of the digital era and personal networks of communication has fundamentally changed the landscape of health communication, with the majority of the population having convenient access to diverse health-related information and an increasing number of people using the Internet to learn about or self-diagnose health concerns [3, 4]. In particular, the use of social media for health-related purposes by individuals as well as professional institutions and government agencies has been prominently noted [5]. In parallel with these miscellaneous developments, however, excessive exposure to digital health information can overly distress certain users, leading to various manifestations of health anxiety such as cyberchondria [6, 7] and illness anxiety disorder [8].

Health anxiety occurs when one experiences obsessive and constant worry about one’s health, characterized by a persistent belief in having a serious illness despite the absence of medical abnormalities [9, 10]. The development of health anxiety can be shaped by genetic, personality, psychological, social, and behavioral vulnerability factors [11–14]. A sizable body of literature has highlighted the important role of online information interaction and engagement in the formation of health anxiety [4, 7, 10, 15]. A commonly observed pattern is that individuals may attempt to relieve anxiety by repeatedly searching for health information, yet such behavior often exacerbates the condition and thus leads to increased health anxiety [16, 17]. Given the limited scope of individual life experiences, most people rarely encounter various potential health risks and crises firsthand. Threat perceptions are typically shaped indirectly through media representations and social platform discussions. Therefore, the nature of digital media content as well as its engagement is pivotal to our understanding of health anxiety and its interventions [Seo HY, Peng W, Jerin SI: Investigating Internet use and patient engagement: the moderating role of patient-centered communication on information overload and health anxiety, forthcoming, 18].

In the current era of personal communication networks, social media has become a key platform for the general public to access health information and share health concerns [19]. Most young adults engage various social media platforms, both through active information seeking and through passive (or what is often called incidental) exposure, and many of them look to social media influencers for health and lifestyle inspiration [20, 21]. An expanding body of literature has demonstrated a significant association between social media engagement and health anxiety. First, the influx of large amounts of health information can cause information overload, heightening users’ cognitive burden and increasing susceptibility to anxiety and panic [22, 23]. Second, social media is rife with unverified health rumors and misinformation, which may exaggerate disease risks and harms, thereby triggering confusion and anxiety [24, 25]. Health information on social media often portrays idealized health standards and lifestyles, and users may perceive their own health as inadequate when engaging in social comparison, thus leading to anxiety and feelings of inferiority [26, 27]. Moreover, people who aggressively seek health information online may not possess the adequate cognitive capacity to process such information but they are inclined to share health information in the form of misconceptions and misinformation on social networks, thus magnifying potential threads and propelling health anxiety [28]. Finally, social media algorithms can expose specific groups to disproportionately negative or misleading health information, thereby exacerbating inequities [29, 30], and information related to health anxiety—such as symptoms, diagnoses, treatments, and personal concerns and experiences—is more likely to spread [31, 32].

Public Twitter (now X) feeds have been utilized to shed light on health anxiety trends for the general public [33, 34] as well as individual self-disclosive symptoms [35]. Likewise, Sina Weibo—China’s Twitter (X) counterpart—has consolidated itself as an eminent venue wherein critical debates and diverse public spheres exist and thrive under certain circumstances [36]. In particular, Weibo has become a pivotal platform for exhibiting and discussing health concerns, especially during moments of unfolding public health crisis and emergencies [5, 22, 37]. Specifically, Weibo chatters were analyzed as a barometer for public attitudes towards anxiety disorders [38–40], collective sharing of public emotions [41], and noninvasive prediction of depression among individual users [42]. Nonetheless, there is a deficiency in extant literature offering a feasible framework and baseline model to view Weibo as a public platform for sharing health anxiety sentiments.

An oft-employed framework in analyzing social media content is the Extended Parallel Processing Model (EPPM), which was initially proposed by Kim Witte to explain how information influences health behavior (e.g., self-efficacy and response efficacy) through the central variable of fear [43]. EPPM provides a useful conceptual structure to examine the threat–efficacy dynamics of social media [44], and it was employed to assess public risk perception [45, 46] and how perceived threads motivated preventive behaviors [47] during the COVID-19 pandemic. While most of EPPM research has been done with surveys and experimental methods, growing efforts have been made with analyzing social media content, as seen in the investigation of health messages on Weibo [48] and Weibo chatters of HPV vaccine [49]. To the best of our knowledge, little research has been conducted to evaluate threat and efficacy dimensions as they relate to health anxiety on social media.

This study therefore embraced two major objectives. First, it aimed to examine health anxiety discussions on Weibo in terms of their topic structure and information characteristics as well as their situated contexts. Second, it sought to dissect health anxiety chatters on Weibo through core concepts of the Extended Parallel Processing Model framework with an emphasis on threat and efficacy thematic elements.

## Methods

### Data collection

Discussion related to “health anxiety” on Weibo was the focus of interrogation. Data was collected via Sina Weibo’s open platform APIs, which are developed to allow third-party application programmers to harvest data points such as post (tweet), repost count, and other metadata. Keyword combination of “health” AND (“worry” OR “concern” OR “anxiety”) was used for the search, which covered the period from January 1, 2021, to December 31, 2024. The dataset included username, post (tweet) content, topic classification, repost count, comment count, like count, and posting time. This search procedure yielded a total of 138,587 Weibo posts for the data corpus.

The Weibo posts (*n* = 138,587) were preprocessed as follows prior to further analysis. First, message cleaning was performed to remove emojis, hashtags, punctuation marks and URLs from each post, retaining only the textual content. Second, short and duplicate posts were retained in this study for the following reasons. Regarding the former, the decision was based on the reasoning that phrase fragments, chatspeak and shorthand expressions are commonly used on Weibo by individuals as a quick way to convey one’s attitude or emotion to specific health anxiety-related scenarios or episodes. Excluding these posts would eliminate a substantial portion of the data and drastically change the overall semantic data structure. For the latter, duplicate Weibo posts were mostly microbloggers’ recirculation of public health-related messages or announcements from health institutions, public service organizations or government bodies, and the reposting (often with added comments) itself is a manifestation of the pulse of public sentiments on hot-spot issues and topics. These are potentially significant external factors that modulate certain individuals’ health anxiety, and each repost can directly reach a different follower base. Removing these duplicated posts would change the holistic scope of the impact of such communications and therefore misrepresent the thematic configuration of the overall Weibo sphere.

Given the time and effort limitations of manual analysis, a sampling approach was adopted to narrow down the list of Weibo posts to be content analyzed. Based on the number of likes, the 2,000 most popular Weibo posts were selected as the data sample. Each of the selected posts was checked by a researcher for relevance, and posts with low relevance to health anxiety, duplicate posts, or those containing only advertisements or videos, were excluded from the sample. This filtering process led to a cleansed set of 1,480 valid posts for further analysis.

### Topic modeling

This study employed the LDA model feature implemented in Python. LDA is an unsupervised machine learning text mining technique, consisting of three levels: words, topics, and documents. It is well-suited to identify latent topics in large document collections or corpora. The process begins with preprocessing raw text into word-frequency vectors, transforming unstructured language into numerical data suitable for statistical modeling. Then, probabilistic techniques are applied to construct multi-level distributional relationships between words and topics, as well as topics and documents, to achieve topic clustering and classification of the text [50, 51].

Posts on health anxiety collected from Weibo (*n* = 138,587) were preprocessed using the built-in modules (i.e., re, itertools, os and sys) in Python’s Standard Library, and stop-word removal and word segmentation procedures combining numpy, pandas, tomotopy, nltk, sklearn, and jieba (specifically developed to tokenize Chinese words) features were utilized to extract top characteristic words. Next, the LLM-based LDA model using tomotopy and Gibbs-sam-pling procedures were employed to generate topic-word matrix and calculate topic intensity values as well as identifying topic models (reported in Table 1).

We adopted a strategy combining “Quantitative Evaluation” and “Qualitative Review” to pinpoint topic models. In the quantitative stage, we evaluated K-value metrics of Perplexity and Topic Coherence, with the former yielding a possible 6-8-10 model while the latter suggesting a 5-6-8 solution. Significant Elbow Points were observed at K = 5 for Topic Coherence and K = 8 for Perplexity. In the qualitative stage, we resorted to “Human Judgment” by employing four trained researchers to compare the 5-6-8 solutions in searching for the best K-model that presents an optimal balance between thematic coherence and health anxiety conceptual alignment. This process combining (initial) statistical recommendation and (final) human interpretation led to a K = 8 optimal solution as presented in Table 1.

Once the number of topics was finalized, the pattern of topic-word distribution was identified under each topic through an LDA process integrating model training and human analysis. First, the words suggested by LDA modeling were reviewed, cross-checked and validated against the related Weibo posts by human coders. Next, relevant topic labels were created by the researchers based on semantic relevance. The topic-word distribution was ordered by word weight. For each topic, the 10 most frequent words were selected as representative keywords. Words within each topic were ranked from highest to lowest frequency. Higher frequency corresponds to a higher position in the list. The results are presented in Table 1.

### Content analysis

Two doctoral students served as coders to process the sampled 1480 posts. To ensure reliability, 5% of the sample was randomly selected for training and reliability testing before remaining posts were processed. The inter-coder reliability across all categories reached 90.06%. Content analytical categories were constructed based on EPPM, including severity, susceptibility, self-efficacy, and response efficacy. The posts were coded to indicate whether the posts mentioned elements within each category.

1. *Severity*. Severity refers to health risks or threat elements in the information, highlighting potential negative consequences. Severity in health anxiety primarily falls into four categories: (a) *emotional threats* – including expressions of negative emotions such as worrying about illness or feeling helpless and depressed during treatment; (b) physical threats – including health deterioration such as declining organ function, reduced physical flexibility, and poorer sleep quality; (c) *social consequences* – such as threats to career development and social relationships (e.g., being unable to work after illness, experiencing academic stagnation, or suspending social activities); (d) *economic threats* – such as medical expenses and income loss. As an example for physical thread (b), one post stated: “After radiotherapy, my throat muscles failed to coordinate, so I could only consume liquid food.” Another post noted: “Due to illness, I went into debt and disrupted the family atmosphere,” reflecting both economic threats (d) and social consequences (c).
2. *Susceptibility*. Susceptibility refers to an individual’s perceived likelihood of experiencing health risks. It is expressed through examples or numerical data: (a) *factual examples*—cases of health anxiety that have already occurred, either personally or in others, such as a blogger documenting their diabetes experience; (b) *hypothetical examples*—narratives of possible future events, such as “The risk of metabolic disorders may emerge within five years after high sugar intake”; (c) *evidence-based research examples*—quantitative data or empirical statistics, such as “Over 300 million people in China suffer from sleep disorders.”
3. *Self-efficacy*. Self-efficacy relates to an individual’s confidence in effectively responding to suggested measures while caught in certain situations of health anxiety. It is mainly expressed in two ways: (a) *building self-confidence*—for example, “Although the disease persists, recovery is possible through treatment and maintaining a positive mood”; and (b) *providing emotional support*—such as advocating a rational and positive attitude toward certain health outcomes: “It is important to avoid excessive worry. Many diseases are not highly threatening and can be alleviated through preventive measures and timely consultation.”
4. *Response efficacy*. Response efficacy pertains to an individual’s perception of how effective the information is in reducing or eliminating threats. It includes three categories: (a) *effectiveness of supporting policies*—support provided by healthcare systems, medical insurance, and state-issued policy measures; (b) *effectiveness of social support* —derived from material or emotional support by partners, parents, relatives, or friends; (c) *effectiveness of technical support*—emphasizing the use of professional knowledge and advance technologies for health recovery, such as fitness and nutrition knowledge.
5. *Information responsiveness*. Based on the EPPM framework, an individual’s attitude toward accepting or rejecting information can be measured by their level of engagement with social media content. On Weibo, social cues generated by system automation and user interaction—such as views, shares, comments, and likes—serve as critical indicators for assessing information communication effectiveness and public response [52, 53]. Specifically, like count reflects perceived value and emotional resonance, functioning as a quantifiable indicator of social support and group endorsement [54, 55]. Therefore, this study employs like counts on Weibo posts as an operational measure of information responsiveness.

## Results

### Topic analysis of Weibo health anxiety chatters

The topic-word matrix for each topic was listed in Table 1. As we explained in the Methods section, the label for each topic was added by the researchers through reading the keywords and digesting samples of Weibo messages. Words were listed by frequency (high to low), and the intensity value (aka topic weight) for each topic projects the probability distribution of this topic-word cluster over the whole dataset of Weibo posts.

**Table 1:** Distribution of topics, keywords and intensity values in health anxiety discussions (*N* = 138,587)

Topic	Keywords	Intensity value
Positive living and health ideals	Everyday life, joy, hope, daily routine, like, livelihood, love, work, healthy body, happiness	0.4456
Sleep disorders and mental health	Sleep, patient, treatment, symptoms, diseases, cause, influence, stress, depression, insomnia	0.2366
Adolescent mental health education and social adaptation	Children, mental health, teenager, pupil, student, psychology, education, school, vitiligo, society	0.1402
Healthy lifestyle and self-management	Small secrets, coffee, swimming, aerobics, small tips, running, drinking more water, medical beauty, cereals, sugar	0.0548
Balance between wealth and happiness	Money, future, exchange, ignore, happiness, lament, rush, recovery, fortune, inactivity	0.0419
Common diseases and pet health	Cats, intestines, myopia, probiotics, leukoplakia, puppy, rejection, credit, defecation, autumn	0.0359
Wellbeing of pregnant women and infants	Baby, mother, pregnancy, postpartum, pregnancy period, pregnancy preparation, breast milk, worry, makeup, milk powder	0.0290
Concerns about health risks	Children’s wellbeing, summer break, idiopathic symptoms, smart watch, binge eating, volunteer, misleading signs, poor people, safe and sound, good luck	0.0143

The first topic, positive living and health ideals (intensity = 0.4456), pertains to health anxiety threads that emphasize maintaining a positive outlook, pursuing happiness, and prioritizing physical health through self-regulation. Keywords such as “daily life,” “happiness,” and “hope” exhibit aspirations for optimal wellbeing, while “physical health,” “love,” and “joy” highlight the link between physical and mental health. While these terms generally portray a positive lifestyle discourse, they are typically used in Weibo messages in the context of our research to reflect a tactical (counter-measure) approach to handling health anxiety pressures and challenges through self-regulation and self-modification of emotional, behavioral and other responses. For example, one post said “After a stroll on such a beautiful day, I am full of hope. Let health anxiety go to hell and let a healthy and happy life stay!” In another Weibo message, the author wrote “Looking around, most people of my age are settling into a peaceful and happy life, with a steady good job, a sweet family and uneventful daily routines. I want to be like them, yet I am not totally satisfied with being one of them. For me, I am full of anxiety – with age, body, appearance, health, and love. I wish life was simpler.” This suggests that some users perceive health as a prerequisite for happiness, emphasizing positive psychological states and envisioning an ideal lifestyle between work and joy, even in the face of anxiety triggers in daily life and work.

Topic 2 (sleep disorders and mental health; intensity = 0.2366) contemplates the intersection between sleep problems (e.g., insomnia) and mental health conditions (e.g., stress, depression), along with their effects on patients and possible treatment options. The following post is most typical of this category:

Does this scenario happen again and again to you? Scroll on the phone, and suddenly it is 2 am; want to go to sleep early but feel night is the only time belonging to you; a chest pain after working a long night, and you order online a long list of health supplements … Look at the top-two trending topics today, they seem to suggest to you that it is time to prioritize your health. Browse online comments, many young people feel burned out through overtime work and are over worried about their health. Are you one of them? Do you have health anxiety?

Topic 3 is adolescent mental health education and social adaptation (intensity = 0.1402) and highlights adolescents’ mental health in school and social contexts, emphasizing the role of education in promoting mental health, addressing challenges such as vitiligo and fostering social adaptation. Keywords such as “children”, “teenager”, “pupil”, and “student” identify the affected groups, while “mental health”, “psychology”, “education”, “school” and “social” indicate the focus on psychological aspects. The following post illustrates this thread well: “Having a sick kid completely drains you out. Maizi [child’s name] just recovered, but then Doudou [name for another child] gets unwell. Dry cough the first day, and high temperature the next. Took him to the doctor, and he screamed like a pig being slaughtered when his blood was drawn … A mother’s health anxiety – sick kids, and fall-behind schoolwork.”

Topic 4 (healthy living and self-management; intensity = 0.0548) covers diverse aspects of healthy living, including vitality maintenance (e.g., coffee consumption), exercise (e.g., swimming, aerobics, running), and balanced diet (e.g., hydration, grain and sugar intake). It also includes medical aesthetics as a modern lifestyle choice, which fits the topical focus on providing practical tips to support everyday health management. Here are two examples of this category. One Weibo post connects health anxiety to a popular teleplay: “Recently, Nothing but Thirty [a teledrama] triggers a new round of health anxiety. Gujia [wife of the main male character] only allows her husband to eat fruits and vegetables for dinner. In fact, our health anxiety will only grow as we grow in age.” Another post links sudden death and health anxiety: “Sudden death has frequented news headlines in the past month. According to statistics, 600 thousand people die of sudden death each year in our country, with 43% of them below 40 … Accelerated pace and mounting pressure in life are turning sudden death into a health anxiety for many. Want to know how to prevent sudden death? Then follow XX.”

Topic 5 (intensity = 0.0419) reveals the complex mentality of individuals facing the trade-off between financial gain and happiness. In pursuing wealth, individuals may neglect or sacrifice health, leading to heightened future-oriented anxiety. Keywords such as “money”, “future”, and “exchange” directly demonstrate this trade-off. Terms such as “ignore”, “lament”, and “rush” reflect contradictory attitudes: neglecting health for success yet regretting its loss and needing recovery at a cost. This post amply shows one’s profound conundrum: “One felt bored and longed to grow up while a child, but he wanted to go back to childhood when he becomes an adult. One squanders his health to gain wealth and then spends his wealth to get his health back. Anxiously looks for the distant future but fails to enjoy the moment.” The other post is more straightforward: “I can’t help but lament while looking at the abnormal numbers on my medical report—it is worth it to make all the money at the sacrifice of my health? If I will be bedridden in the future, does it really matter how much money I can make? This health anxiety can be costly.”

Topic 6 (common diseases and pet health; intensity = 0.0359) reflects concerns about personal and pet health, particularly as related to digestive, vision, and skin-related issues. Keywords in this category indicate interest in gut health and microbiota balance, likely linked to the rise of probiotic products and suggested remedial actions. “Myopia” and “leukoplakia” point to concerns about prevalent health problems, and the inclusion of pet-related terms such as “cats” and “puppy” shows that pet health has become part of health anxiety and owners closely link pet health to family well-being. Even though some posts mentioned pet illness and human disease separately, many mixed them together, often as a way to reflect mutual care and mutual dependence of human-pet relationships. This post serves as an example: “While buying probiotics for dietary regulation for myself today, I picked some for my dog as well. I am on a special diet, and it eats prescription food. Seeing the dog’s struggles, along with my own discomfort, my health anxiety immediately gets magnified!”

Topic 7 (intensity = 0.0290) focuses on maternal and infant health issues, showing the concerns and anxieties of mothers regarding themselves and their children. Keywords in this list encompass the maternal journey from pre-pregnancy to postpartum recovery, alongside infant growth. “Breast milk” and “milk powder” affirm infant feeding methods as a frequent source of postpartum anxiety. Being pregnant puts one in a constant state of anxiety over an extended list of things, as shown in this example from a post by an expectant mother: “Everyday is a day of anxiety—anxiety over not being able to wear my favorite dress, anxiety about adding to my waistline, anxiety over how long this vomiting lasts, anxiety about the health of the fetus, anxiety over if my emotional fluctuations will effect the little one inside. Since day one of my pregnancy, anxiety surpasses happiness.”

Finally, topic 8 (intensity = 0.0143) captures varying types of anxiety about uncertain health risks across multiple domains ranging from how one’s child spends the summer to binge eating. It typically covers the type of social media bantering one may post based on spontaneous feelings of the moment, as seen in this example: “Since wearing my Xiaommi watch, I have developed health anxiety. It often shows my blood oxygen as 90 and puts my heartbeat at 140. Turned out these are due to errors of inaccurate reporting. Paid money to get this unwarranted anxiety.” Keywords such as “idiopathic” and “gluttony” reflect concerns over atypical or unexplained health conditions, and the keyword “smart watch” relates to trending wearable health devices, highlighting public attention to technological interventions while also implying anxieties about dependence and privacy. “Misleading signs” and “poor people” reveal concerns about misinformation, resource accessibility, and social equity. Expressions like “safe and sound” and “good luck”, which often are mentioned in association with health worries, serve as well-wishing felicitations and provide emotional buffering and psychological comfort.

LDA-based topic analysis revealed several thematic patterns of health anxiety discussions on Weibo. Positive living and health ideals is by far the most dominant thread, suggesting that despite anxiety, users lean toward psychological adjustment aiming for positive outcomes in everyday living. The rest of the topics (2–8) ranges from individual physiological issues (e.g., sleep, digestion) to broader social concerns (e.g., identity, economics, intergenerational responsibility), testifying to the multi-dimensional nature of health-related anxiety. Health anxiety among specific groups—such as adolescents (topic 3), pregnant women (topic 7), and pet owners (topic 6)—forms distinct threads, reflecting trends of “contextualization” and “group differentiation” in social media health communication. Healthy living and self-management, which is behavior-oriented and highly practical, is ranked relatively low (4th) in intensity, suggesting that health anxiety on Weibo has not yet developed a prominent action-based focus.

### EPPM information characteristics

The results of the EPPM-based content analysis are presented in Table 2. Health threat information frequently appears in social media discussions. The most common type concerns the severity of health threats (73.24%, *n* = 1084), followed by susceptibility information (51.55%, *n* = 763). Health efficacy information is rather limited, with response efficacy least mentioned (16.15%, *n* = 239) and self-efficacy second (25.41%, *n* = 376). Overall, threat and efficacy elements are quite evenly distributed in health discussions. Weibo posts emphasize health risks—particularly severity and susceptibility—while sporadically offering measures or contemplations to mitigate them. Detailed results are shown in Table 3.

**Table 2:** Information elements in Weibo health anxiety discussion (*N* = 1480)

Conceptual Element		Yes (mentioned)	No (not mentioned)
Threat	Severity	1084(73.24%)	396 (26.76%)
	Susceptibility	763 (51.55%)	717 (48.45%)
Efficiency	Self-efficacy	376 (25.41%)	1104 (74.59%)
	Response efficacy	239 (16.15%)	1241 (83.85%)

**Table 3:** Information characteristics in Weibo health anxiety discussions (*N* = 1480)

Information element	Index	Yes (% mentioned)
Severity (*n* = 1084)	Emotional threats	680(62.73%)
	Physical threats	311(28.69%)
	Social consequences	257(23.71%)
	Economic threats	109(10.06%)
	Others	45(4.15%)
Susceptibility (*n* = 763)	Factual examples	489(64.09%)
	Hypothetical examples	226(29.62%)
	Evidence-based research examples	58(7.60%)
Self-efficacy (*n* = 376)	Building self-confidence	146(38.83%)
	Emotional support	264(70.21%)
	Others	11(2.93%)
Reaction efficacy (*n* = 239)	Effectiveness of health supporting policies	9(3.77%)
	Effectiveness of social support	198(82.85%)
	Effectiveness of technical support	27(11.30%)
	Others	12(5.02%)

#### Severity

Severity information is frequently mentioned and features prominently in users’ health threat concerns, present in 73.24% (1084 out of 1480) of the messages. Further analysis shows multidimensional expressions of severity, with emotional threats (62.73%, *n* = 680) at the core. Users emphasize the psychological impact of health problems through negative emotions (e.g., “Staying up late makes me fear sudden death every day”). The salience of emotional threats suggests that users focus more on subjective feelings than on objective physiological indicators, often resorting to emotional support and resonance to alleviate stress, loneliness and other struggles. Physical threats (28.69%, *n* = 311) come next on the list, focusing on direct physiological damage and consequences (e.g., “Obesity can lead to hypertension, diabetes, and body collapse”). Notably, physical and emotional threats are often intertwined. For example, pain descriptions convey both physical discomfort and negative emotions such as anxiety and fear. Social consequences (23.71%, *n* = 257) and economic threats (10.06%, *n* = 109) extend health threats to social disruption (e.g., “being isolated after illness”) and financial burden (e.g., “cancer treatment bankrupts families”). Additionally, 4.15% (*n* = 45) mentioned other threats, such as unequal medical resources and lagging technology.

To summarize, severity information as displayed in Weibo health anxiety discussions reveals emotional, physical, and social characteristics. Users may amplify urgency through emotional catharsis, combined with physical narratives and descriptions of economic/social consequences.

#### Susceptibility

Susceptibility information is widely cited as concerns about personal health risks, found in 51.55% (763 out of 1480) of the posts. Further analysis shows factual examples as dominating susceptibility expressions (64.09%, *n* = 489). Users anchor risks in real cases (e.g., “A friend had a myocardial infarction after staying up late” or “A colleague found a thyroid nodule during a checkup”) or personal experiences to enhance credibility. Hypothetical examples (29.62%, *n* = 226) link risks to behavior using “if…then” logic (e.g., “If you sit too long, lumbar disc herniation will occur”), stimulating prevention motivation, while evidence-based scientific examples are much less common (7.60%, *n* = 58). Weibo posters generally favor factual and hypothetical examples over scientific (evidence-based) ones when discussing susceptibility. This suggests a stronger focus on personal experience and emotional resonance than on research evidence.

#### Self-Efficacy

Self-efficacy information is less frequently mentioned, with 25.41% (376 out of 1480) messages displaying pertinent discussions. Our sub-category analysis points to the domination of emotional support (70.21%, *n* = 246) in this overall category. Users build efficacy through emotional connections, sharing mutual assistance (e.g., “Joining a sports group kept me going”), offering comfort (e.g., “Don’t worry, I’ll go with you”) or citing group successes (e.g., “Our weight-loss camp succeeded, so anyone can”). Building self-confidence (38.83%, *n* = 146) typically relies on affirming individual ability to gain the upper hand over health challenges. Users are inclined to place emphasis on sharing personal experiences (e.g., “I cured insomnia by diet adjustment”), listing action steps (e.g., “Run 30 minutes daily for 3 months”), or citing authoritative advice (e.g., “My doctor said exercise sped up recovery”). Health anxiety discussions often center on emotional support, as emotional messages tend to solicit more positive and supportive feedback from the audience on Weibo. By contrast, self-confidence building requires active self-adjustment and calls for intentional effort to actualize, thus likely making it less appealing in terms of user engagement on Weibo.

#### Response efficacy

As seen in Table 2, response efficacy is the least mentioned out of the four categories (found in 16.15% or 239 out of 1480) posts. When it is mentioned, it is typically focused on the enabling role of social support systems in health behavior improvement (82.85%, *n* = 198). Weibo posters emphasize social networks through examples: family support (e.g., “Quitting drinking succeeded with family help”), community assistance (e.g., “Patient groups helped me with avoiding pitfalls”), and institutional guidance (e.g., “Hospital courses taught scientific eating”). Fall less mentioned is technical support (11.30%, *n* = 27), which pertains to the role of using digital aids such as fitness apps (e.g., “Training lessons through Keep [a fitness app] sustained my exercising”) and monitoring devices (e.g., “Smart bracelets reminded me to regulate heart rate”). Policy support effectiveness, which is rarely cited (3.77%, *n* = 9), mainly acknowledges macro issues (e.g., insurance coverage, public services) and is typically expressed critically (e.g., “Exams are too costly; policy support is insufficient”).

#### Audience engagement with threat and efficacy information

Based on the coding of Weibo posts containing health anxiety threat and efficacy messages, associated like counts were utilized as an indicator of information responsiveness/engagement. Correlation analysis examined the impact of threat and efficacy information on audience engagement in health anxiety discussions. As shown in Table 4, severity information showed a significant negative correlation with likes (*r*=-.077, *p*<.01), and susceptibility information also demonstrated a significant negative correlation (*r*=-.054, *p*<.05). Conversely, self-efficacy information exhibited a significant positive correlation with likes (*r*=.104, *p*<.001), as did response efficacy information (*r*=.145, *p*<.001). These results indicate that higher perceived health threats in health anxiety discussions correlate with fewer post likes, whereas higher perceived efficacy correlates with greater engagement. In summary, information containing efficacy elements is more likely to be positively responded to.

**Table 4:** Correlation analysis of thread and efficacy information and audience engagement

	Severity	Susceptibility	Self-efficacy	Response efficacy
Like counts	− 0.077**	− 0.054*	0.104***	0.145***

*p < .05, **p < .01, ***p < .001

## Discussion

The concept of “health anxiety” has evolved beyond its original pathological scope and has attracted a cumulative body of research from diverse disciplines [9, 56–59]. Within online social networks, individuals claiming to experience health anxiety encompass a broad segment of the general public [7, 10, 57]. Most lack clinical diagnosis but develop strong anxiety or fear regarding health due to personal circumstances, socio-cultural influences and the omnipresent online information environment [10, 60]. The popularization of social media in everyday life is reshaping the generative logic and communication pathways of health anxiety [15, 61].

This study aimed to systematically investigate topic structure and information characteristics of health anxiety discussions on Sina Weibo through the lens of the EPPM framework. Our research on Weibo chatters reveals that health anxiety has established a pervasive presence, encompassing a wide range of thematic threads. The range of topics spans from individual physical issues to social concerns and specific subgroup health challenges. As a demonstration of the multiplex nature of health anxiety, physical, psychological, and social dimensions have been identified in the topical semantics. Areas of health concerns comprise sleep disorders, mental health, common diseases and pet health, while adolescents, pregnant women, baby and school-age children are singled out as targeted subgroups of health anxiety. The multidimensionality of health anxiety on social media suggests that the primary focus on excessive worry over personal health in the conventional diagnostic standards by the medical sciences [9, 10, 62] may be too narrow, and new conceptual and clinical definitions may be needed in order to address the full scope of related symptoms and tendencies.

Of the eight major topical threads the LDA modeling detected, it is worth highlighting that leading the rest by far is a constructive spin on uplifting health outcomes through positive lifestyles and ideal goal setting when facing health-related threats and uncertainties. Resonating with the dominant modality of staying positive, health anxiety discussions delve quite a bit into proactive measures and approaches in handling specific challenges through education, social adaption, pro-health living styles, and self-management via food and physical exercise. In particular, there is also notable deliberation on the existential meaning of life in the context of balancing between wealth accumulation and health gains as well as intergenerational care. This suggests that health anxiety on social media goes beyond merely illness fear and extends beyond the welfare of oneself, and it incorporates aspects of lifestyle aspiration, emotional self-regulation and the pursuit of ideal wellbeing.

Informational element analysis reveals a distinct imbalance between health threats and health efficacy. Health threats dominate as the primary information element, while health efficacy elements are significantly underrepresented. User perception of health anxiety shows a contradictory pattern of “high threat vs. low efficacy”—health threat information appears far more frequently than efficacy information. This imbalance may reflect users’ heightened concern about health risks and uncertainty. However, an overemphasis on threats can cause information overload and intensify health anxiety [Seo HY, Peng W, Jerin SI: Investigating Internet use and patient engagement: the moderating role of patient-centered communication on information overload and health anxiety, forthcoming, 63]. According to the EPPM framework, severity and susceptibility are key components of threat-related risk perception, and these cognitive factors significantly contribute to anxiety [43]. When perceived efficacy exceeds perceived threat, individuals show greater motivation to control danger, and persuasive messages are more readily accepted [63]. Findings in this research are consistent with the EPPM model, wherein threat and efficacy elements in health anxiety information prompt individuals to assess perceived severity and susceptibility to health threats. Specifically, when health information effectively activates an individual’s coping efficacy (e.g., self-efficacy and response efficacy), they are more likely to engage in risk-control processes, develop protective motives and adopt adaptive behaviors such as updating health beliefs or implementing preventive measures to proactively manage potential health hazards. Conversely, when perceived threat is excessive and individuals lack adequate coping resources, they are more likely to engage in fear-control processes, exhibiting behaviors such as information avoidance, risk denial, or emotional suppression—responses representing maladaptive coping strategies.

Furthermore, individual engagement with health threat information is significantly moderated by their subjective psychological perceptions. As far as information engagement (measured by like counts) is concerned, correlational analysis indicates that audience endorsement is negatively associated with threat intensity in health information but positively correlated with efficacy intensity. This suggests that Weibo users may be more inclined to engage with health efficacy information while they tend to steer away from information that emphasizes health threats exclusively. When confronted with health information that may trigger anxiety, individuals tend to seek psychological safety and a sense of control. This suggests that, in information environments presenting risks and uncertainties, incorporating action-based and feasible coping strategies may alleviate individual fears, enhance information persuasiveness, and increase audience engagement, thereby improving health communication effectiveness. Nonetheless, it is worth pointing out that the statistical association is low in the findings and this pattern of interaction bears testing out in future research.

In contemporary society, certain forms of anxiety can be classified as “vicarious anxiety” in that they are rooted not in direct personal experiences of real threats but rather in psychological states shaped by social communication processes—the experience of anxiety in observing others expressing it [64, 65]. Recurring narratives of health threats in online discussions as extrapolated through topic modeling and content analysis from this study may provide a pivotal basis for understanding the contemporary collective cognitive landscape of health anxiety in China. Our information element analysis of Weibo messages reveals that susceptibility perceptions in health anxiety discussions are often grounded in factual or hypothetical cues. The anxiety individuals experience often stems from secondhand accounts of others’ experiences or subjective inferences about potential health consequences rather than from their own immediate health crises. These vicarious third-party experiential narratives have the potential to trigger individuals’ imaginations of health threats and thus foster an anxiety mechanism based on cognitive projection rather than actual risks. How the dynamics in such an information ecosystem contribute to the development of individual health anxiety warrant further scrutiny in future research.

## Limitations

The findings of this study are best understood in the context of its limitations. First of all, because the data driving the analysis in this research comprises textual content collected on Sina Weibo, no causal inferences can be made regarding the impact of the text on users. User perceptions and responses to Weibo messages are important questions that should be addressed by engagement data through users directly. In this regard, the EPPM-related perspectives could benefit from corroboration using survey and experimental designs. Second, the time frame for Weibo data covers the 2021–2024 period in the wake of the COVID-19 pandemic. Indeed, the aftermath of the outbreak very much lingered on in 2021 and 2022, and its impact on the general public is very much a compounding factor in online chatters. To what extent the COVID-19 global health crisis has figured in individual manifestations of health anxiety as well as how this has influenced Weibo discussions cannot be ascertained. Finally, due to the condensed nature of Weibo communications, the keyword searches may miss posts that are directly relevant to the research topic on one hand, and they may include messages that are not closely pertinent to our research interest on the other hand. Collapsing all Weibo posts into one data corpus and feeding that into a single data pipeline may ignore important contextual clues that are central to message interpretation or lead to deviations from deep message themes hard to be captured through automated text analysis. Additionally, the Weibo posts sampled based on number of likes for content analysis may yield an inaccurate representation of the overall data and hence result in a biased projection of the overall results.

## Conclusions

Various thematic aspects of health anxiety-related discourse are prevalent in the Weibo discursive sphere. Major topic threads cover a range of issues from sleep disorders, adolescent mental health, healthy lifestyle to common diseases and pet ailment, which is a testament to the prevailing areas of concern to the general populace in Chinese society. The multidimensional nature of health anxiety finds robust manifestations in the multiple physical, psychological, and social layers of Weibo discussions in relation to diverse issues and settings. Moreover, meaning-of-life deliberations are also found in the context of wealth accumulation vis-à-vis health gains. Subgroups that have been featured prominently are school-age adolescents, pregnant women, infants, cats and dogs, showing that these are the sections prone to be either victims or causes of health anxiety. Of particular note, dominating the thematic discussions is a thread positively constructed centering on uplifting, confidence-building lifestyles and goal setting when facing health-related risks and uncertainties. There is also plentiful message-sharing oriented towards proactive measures and actionable approaches to come to grips with specific challenges through education, social adaption, food and physical exercise. The findings highlight that health anxiety on social media is collectively constructed through a mixture of threat narratives, lifestyle ideals, emotional support and episodic coping insight. At the theoretical level, they suggest that health anxiety online could be more fruitfully conceptualized beyond the narrow focus found in established clinical definitions.

There is a distinct pattern of “high threat vs. low efficacy” contradiction among users’ conception of health anxiety in Weibo, as health threat information gets far more mentions than efficacy information. Findings lend support to the EPPM framework through the interplay of threat susceptibility and self-efficacy and more importantly, the evidence expands theoretical applicability of the EPPM perspective in social media contexts and offers empirical support for understanding how individuals respond to anxiety-inducing information. It illustrates the dual effect of anxiety-inducing information: while it may provoke fear-driven defensive avoidance if emphasis is placed on severity of thread, it can also enhance audience efficacy and hope by stressing actionable coping strategies. These findings have significant implications for health communication professionals and healthcare workers in designing health campaigns and tailoring information needs to targeted demographics and risk groups. When threat information reigns and efficacy clues are sparse, it is advisable that health communication professionals balance risk awareness with coping strategies, emotional support and evidence-based medical guidance to the general public.

## Data Availability

The dataset for this study was obtained from publicly accessible posts on Weibo via the API. Details of data collection and preprocessing are provided in the paper. The data as well as the analytical procedures that support the findings of this study are available from the corresponding author upon reasonable request.

## Abbreviations

LDA: Latent Dirichlet allocation
EPPM: extended parallel process model
